# Identification of cuproptosis-related subtypes, cuproptosis-related gene prognostic index in hepatocellular carcinoma

**DOI:** 10.3389/fimmu.2022.989156

**Published:** 2022-09-13

**Authors:** Lei Ding, Wei Li, Jili Tu, Zhixing Cao, Jizheng Li, Haiming Cao, Junjie Liang, Yiming Liang, Qiangfeng Yu, Gencong Li

**Affiliations:** ^1^ The Second Department of General Surgery, Zhuhai People’s Hospital (Zhuhai Hospital Affiliated with Jinan University), Zhuhai, China; ^2^ Department of Pathology, Zhuhai People’s Hospital (Zhuhai Hospital Affiliated with Jinan University), Zhuhai, China

**Keywords:** cuproptosis, hepatocellular carcinoma, Molecular subtype, immunotherapy, tumor microenvironment, overall survival

## Abstract

Cuproptosis is a novel form of cell death, correlated with the tricarboxylic acid (TCA) cycle. However, the metabolic features and the benefit of immune checkpoint inhibitor (ICI) therapy based on cuproptosis have not yet been elucidated in Hepatocellular carcinoma (HCC). First, we identified and validated three cuproptosis subtypes based on 10 cuproptosis-related genes (CRGs) in HCC patients. We explored the correlation between three cuproptosis subtypes and metabolism-related pathways. Besides, a comprehensive immune analysis of three cuproptosis subtypes was performed. Then, we calculated the cuproptosis-related gene prognostic index (CRGPI) score for predicting prognosis and validated its predictive capability by Decision curve analysis (DCA). We as well explored the benefit of ICI therapy of different CRGPI subgroups in two anti-PD1/PD-L1 therapy cohorts (IMvigor210 cohort and GSE176307). Finally, we performed the ridge regression algorithm to calculate the IC50 value for drug sensitivity and Gene set enrichment analysis (GSEA) analysis to explore the potential mechanism. We found that cluster A presented a higher expression of FDX1 and was correlated with metabolism, glycolysis, and TCA cycle pathways, compared with the other two clusters. HCC patients with high CRGPI scores had a worse OS probability, and we further found that the CRGPI-high group had high expression of PD1/PDL1, TMB, and better response (PR/CR) to immunotherapy in the IMvigor210 cohort and GSE176307. These findings highlight the importance of CRGPI serving as a potential biomarker for both prognostic and immunotherapy for HCC patients. Generally, our results provide novel insights about cuproptosis into immune therapeutic strategies.

## Introduction

Liver cancer is one of the leading causes of cancer-related death in the world, with an appraised incidence of more than 1 million cases by 2025, and about 85% of liver cancer cases are hepatocellular carcinoma (HCC) (1, 2). The initiation of HCC is associated with chronic inflammatory change, which is induced by viral infections, metabolic alterations, etc. (3, 4). Nonalcoholic fatty liver disease (NAFLD) is a typical example of liver metabolism dysfunction increasing susceptibility to HCC, as changes in the metabolic microenvironment persistently exist (5–9). The progression of HCC is driven by metabolic reprogramming, for it shifts the metabolism toward promoting tumor growth and proliferation. Notably, the tricarboxylic acid cycle (TCA) is the key metabolic pathway and connects three major metabolic nutrients (glucose, lipid, and protein) (10). Cancer cells prefer glycolysis to oxidative phosphorylation to fulfill their excessive energetic demand for hyper-proliferation, which is known as the Warburg effect (11). In addition, many cancer cells prefer glutamine to synthesize amino acids and nucleotides. Glutamine is also an essential carbon source for the TCA (12). A previous study found that disturbing the metabolism process of detoxification enzymes (for example, cytochrome P450 2E1 (CYP2E1)) could result in cellular DNA damage and hepatocyte death (13). Cuproptosis, also called copper-induced cell death in a recent study published in Science, facilitated the aggregation of protein lipoylation associated with TCA’s mitochondrial enzymes. However, we know little about the correlation between the metabolism of copper and the progression of HCC.

Systematic therapy for HCC is challenging for minor prolongation of overall survival (OS), including molecular targeted drug therapy (for example, sorafenib, lenvatinib, and regorafenib), and chemotherapy such as FOLFOX4 (combination of Oxaliplatin, 5-fluorouracil, and leucovorin) (14). In recent years, progress has been made in immune checkpoint inhibitor (ICI) for HCC, including cytotoxic T lymphocyte-associated protein 4 (CTLA-4) inhibitors (for example, Ipilimumab), programmed cell death protein 1 (PD-1) inhibitors (for example, Nivolumab, pembrolizumab), and programmed death ligand 1 (PD-L1) inhibitors (for example, atezolizumab) (15–17). The combination of atezolizumab plus bevacizumab (T+A) has gradually become the new front-line treatment for HCC (18). Studies found that targeting and reprogramming metabolism could enhance tumor immunotherapy (19, 20). Previous studies demonstrated that patients with high PD-1/PD-L1, high TMB, high MSI, or low TIDE tend to be more sensitive to anti-PD-1/PD-L1 therapies (21–24). However, due to the genetic, metabolic, and inflammatory heterogeneity of HCC, the traditional molecular classifications have limitations for the identification of the ICI treatment benefit population. Thus, an effective indicator for prognostic and immunotherapy responsiveness considering metabolism is urgently needed.

In this study, we aimed to explore the metabolic features and construct a biomarker based on cuproptosis-related genes (CRGs) for HCC patients, which could both predict the prognosis and immunotherapy response. We explored genetic alterations and the correlation between transcriptional expression and the prognosis of 10 CRGs in HCC. HCC patients were divided into three cuproptosis-related subtypes based on 10 CRGs by consensus clustering. We identified the metabolic features, OS, tumor microenvironment (TME), tumor immune dysfunction and exclusion (TIDE), microsatellite instability (MSI), and tumor mutational burden (TMB) among three subtypes. Moreover, we established a novel prognostic score called “cuproptosis -related gene prognostic index” (CRGPI) to predict OS and ICI responses. Particularly, Decision curve analysis (DCA) proved that the CRGPI classification performed a great clinical net benefit compared with other molecular classification strategies. We further validated the predictive ability of immunotherapy response based on CRGPI in two anti-PD1/PD-L1 therapy cohorts (IMvigor210 cohort and GSE176307). Interestingly, we observed a potential correlation between cuproptosis subtypes C and CRGPI-high subgroups. Our results implied that CRGs could serve as a potential prognostic predictor for OS and responses to immunotherapy, and may offer novel insights into cancer treatment for HCC patients.

## Materials and methods

### Dataset collection and processing

We conducted a mutational analysis of 10 cuproptosis-related genes (CRGs) by 372 HCC samples with cBioportal Liver Hepatocellular Carcinoma TCGA, PanCancer Atlas (https://www.cbioportal.org/). mRNA expression levels of 15 HCC cohorts were obtained by HCCDB (http://lifeome.net/database/hccdb/home.html), including TCGA-LIHC, ICGC-LIRI-JP, and 13 GEO datasets (GSE22058, GSE25097, GSE36376, GSE14520, GSE10143, GSE9843, GSE19977, GSE46444, GSE54236, GSE63898, GSE43619, GSE64041, and GSE76427). Next, four cohorts (GSE14520, GSE76427, TCGA-LIHC, and ICGC-LIRI-JP) with complete clinical information were included for further analysis. To perform the consequent consensus clustering, an HCC meta cohort (GSE14520, GSE76427, and ICGC-LIRI-JP) was integrated, and removed batch effects *via* the “Combat” algorithm. Then, we used the TCGA-LIHC cohort as an external validation dataset.

### Consensus clustering of CRGs

We used the R package of ConsensusClusterPlus to calculate how frequently HCC samples were grouped by 10 CRGs (FDX1, LIAS, LIPT1, DLD, DLAT, PDHA1, PDHB, MTF1, GLS, CDKN2A). And we used the proportion of ambiguously clustered pairs (PAC) to accurately estimate the optimal cluster number (K) (25). Three clusters were identified, and further survival analysis was conducted by the Kaplan-Meier curve with the log-rank test. In addition, we also validated the results of three clusters in the external TCGA-LIHC dataset. In addition, principal component analysis (PCA) was performed by the “ggplot2” R package.

### Pathway enrichment analysis of cuproptosis subtypes

The “GSVA”, “clusterProfiler”, and “Limma” packages were applied for differential expressed genes and pathway enrichment analysis. Besides, we used the gene sets of “c2.cp.kegg.v7.1.symbols” *via* the MSigDB database downloaded on the GSEA website (https://www.gsea-msigdb.org/gsea/downloads.jsp). Under the criterion of |log2(fold change) | > 0.2 and adjust P value < 0.05, we considered it as statistically significant pathways.

### Comprehensive analysis of TME, immune checkpoints, TMB, MSI, and TIDE of three cuproptosis subtypes

To explore the TME of different cuproptosis subtypes in HCC samples, we estimated the proportion of 22 immune cell infiltration by the CIBERSORT algorithm. The gene expression feature profile of the 22 immune cells was downloaded on the website (https://cibersortx.stanford.edu/). We further calculated the ImmuneScore, StromalScore, and EstimateScore for three cuproptosis subtypes by using the ESTIMATE algorithm. In addition, to better understand the potential benefit of ICI, we evaluated the correlation between three cuproptosis subtypes and multiple ICI efficacy-predictive biomarkers (including PD-1, PD-L1, TMB, MSI, and TIDE).

### Construction of the CRGPI

First, we found that 4 genes were associated with OS by performing univariate Cox analysis. Then, we calculated the CRGPI for every HCC patient as follows: CRGPI = Σ(Expi * coefi). Coefi and Expi represented the coefficient and expression of each gene, respectively. To evaluate whether the CRGPI score was an independent prognostic factor, we compared it with other available clinicopathological factors, such as age, gender, and stage. We also evaluated the model performance by the area under the curve (AUC) values for 1-, 2-, and 5-year survivals. Additionally, we divided HCC samples into the CRGPI-high and CRGPI-low groups based on the median score to perform the Kaplan–Meier survival analysis. Similarly, we also validated the predictive power of CRGPI in an HCC meta cohort and ICGC-LIRI-JP cohort.

### Comprehensive analysis of prognosis and immunotherapy response prediction of CRGPI

We compared the CRGPI with four published molecular classifications (Liang et al.; Baohui Zhang et al.; Du et al.; Zhen Zhang et al.) by conducting Decision Curve Analysis (DCA), which could assess the utility of different models for decision-making (26–30). Additionally, two anti-PD1/PD-L1 inhibitor cohorts (IMVigor 210 and GSE176307) were used to demonstrate the predictive value of immunotherapy response of CRGPI. IMVigor 210 includes metastatic urothelial bladder cancer treated with atezolizumab (PD-L1 inhibitor). It has relatively complete mRNA data, OS information, and immunotherapy response information. The R package of IMvigor210CoreBiologies was used to explore IMVigor 210. GSE176307 contains 90 bladder cancer patients treated with pembrolizumab or atezolizuma.

### Drug susceptibility and KEGG analysis

Based on the expression data of the HCC patients, we used the R package oncoPredict for predicting drug response and biomarkers. Through this update method, we could calculate drug sensitivity to find drug-specific biomarkers, predict clinical drug response, and explore the correlation between predictions and clinical features (31) We used the Kyoto Encyclopedia of Genes and Genomes (KEGG) pathway enrichment analysis to evaluate the active pathways in HCC tissues compared to adjacent normal tissues.

### Statistical analysis

Wilcoxon and Kruskal-Wallis test were used for two groups and three groups, respectively. The Kruskal-Wallis test evaluated differences among the three groups. Kaplan–Meier survival plot was assessed by the log-rank test. All analyses and graphics were conducted in R (version 4.2.0, https://www.r-project.org/). p<0.05 was considered significant.

## Results

### Genetic alterations and transcriptional expression of CRGs in HCC

**Figure 1:**
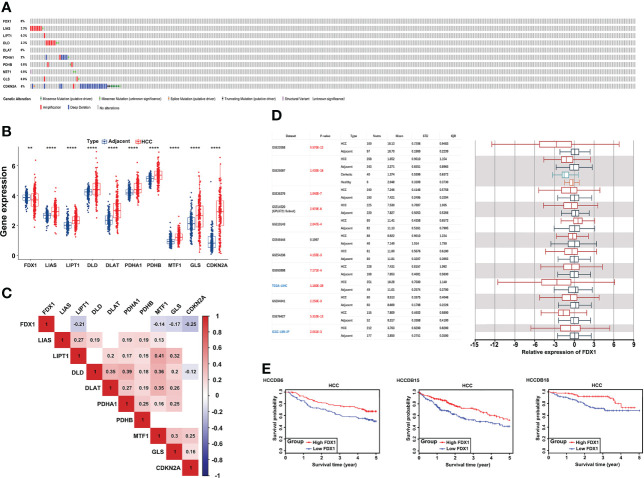
Genetic alterations and transcriptional expression of CRGs in HCC. **(A)** Mutation frequencies of 10 CRGs in 353 TCGA-LIHC samples. **(B)** mRNA expression of 10 CRGs between 177 adjacent and 212 HCC tissues from the ICGC-LIRI-JP cohort. **(C)** The correlation of 10 CRGs in TCGA-LIHC. Red brick represents positive correlation, blue represents negative positive and the depth of the color represents the strength of the correlation between them. **(D)** The expression of FDX1 in 12 HCCDB databases. **(E)** Kaplan−Meier plot for the expression of FDX1 and OS in HCCDB6, HCCDB15, and HCCDB18. CRGs, cuproptosis-related genes; TCGA, The Cancer Genome Atlas; LIHC, liver hepatocellular carcinoma; OS, overall survival. **p < 0.01; ****p < 0.0001.

First, 53 (15.01%) of the 353 TCGA LIHC samples had mutations in the 10 CRGs (FDX1, LIAS, LIPT1, DLD, DLAT, PDHA1, PDHB, MTF1, GLS, CDKN2A). Among them, CDKN2A had the highest mutation frequency (2.55%) and the highest deep deletion rate (5.67%), while LIAS (1.98%) and DLD (1.7%) had a higher amplification, and FDX1 did not have any mutations or CNV **(** **Figure 1A** **).** Since the ICGC database had relatively complete clinical information and large patient samples, it was applied to examine the mRNA expression of 10 CRGs in HCC. Except FDX1 was significantly downregulated, the other nine genes were elevated in HCC tumor tissues compared to adjacent samples **(** **Figure 1B** **)**. Interestingly, with CNV loss or mutation, CDKN2A was expressed at a higher mRNA expression level in HCC samples, suggesting that CNV might not be the only factor involved in regulating mRNA expression. Subsequent spearman’s correlation analysis was performed to explore the correlation of these 10 CRGs. Our study showed similar results to previous studies that FDX1 may negatively regulate the expression of MTF1, GLS, and CDKN2A (32). In addition, the lipoic acid (LA) pathway-related genes and PDH complex revealed an internally positive correlation **(** **Figure 1C** **)**. Since FDX1 was known as a key regulator of copper ionophore–induced cell death, we further examined its expression in HCCDB databases, which included 10 GSE datasets and a TCGA-LIHC dataset. The results of multiple databases further confirmed the lower expression of FDX1 in HCC tissues **(** **Figure 1D** **).** Survival analysis showed that higher FDX1 expression correlated with better OS of HCC patients in GSE14520, TCGA-LIHC, and ICGC-LIRI-JP **(** **Figure 1E** **).**

### Identification of cuproptosis subtypes

To fully understand the cuproptosis expression subtypes of CRGs, the HCC meta cohort (GSE14520, GSE76427, and ICGC-LIRI-JP) was integrated to explore the correlation between subtypes and OS internally, and TCGA-LIHC was used to externally validate the results **(** **Figure S1A** **).**

**Figure 2:**
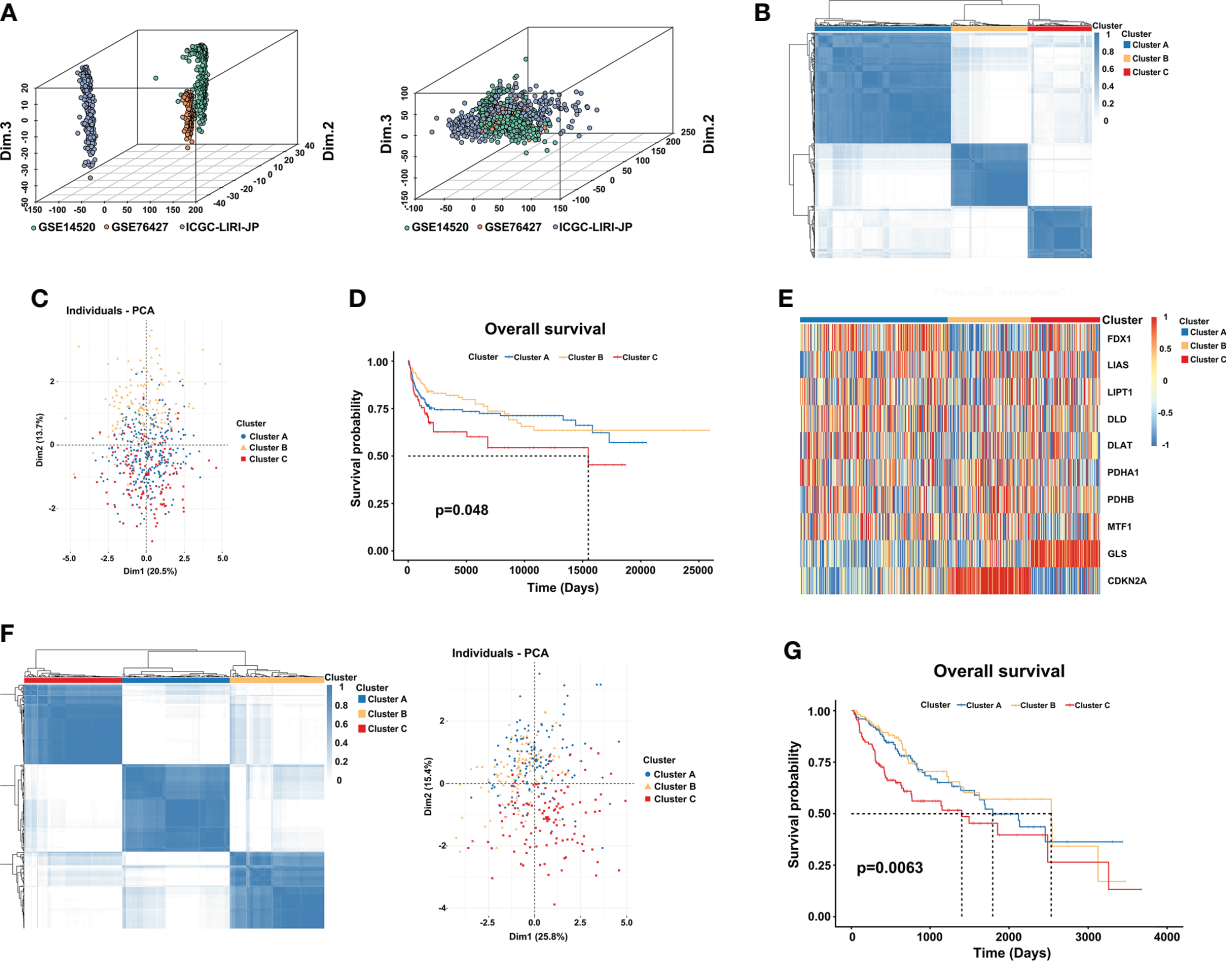
Three distinct cuproptosis subtypes are divided by consensus clustering. **(A)** Constructing an HCC meta cohort by removing the batch effects from GSE14520, GSE76427, and ICGC-LIRI-JP. **(B)** Three clusters (k = 3) were identified by the consensus matrix heatmap in the HCC meta cohort. **(C)** PCA analysis displayed a significant difference between the three clusters. **(D)** Kaplan-Meier survival analysis of OS showed differences among the three clusters (log-rank test, p = 0.048). **(E)** Unsupervised clustering of 10 CRGs in three clusters. Red and blue represent a high and low expression of genes respectively. **(F)** The consensus matrix heatmap also defines three subtypes in the TCGA-LIHC cohort. **(G)** Kaplan–Meier curves with three cuproptosis classes were validated in the TCGA-LIHC cohort (log-rank test, p = 0.0063). CRGs, cuproptosis-related genes; TCGA, The Cancer Genome Atlas; LIHC, liver hepatocellular carcinoma; OS, overall survival.

First, we used the “Combat” algorithm to remove batch effects in the HCC meta cohort **(** **Figure 2A** **)**, and we excluded patients without survival information. Then, a total of 527 HCC patients were included in the further analyses. We used the unsupervised consensus clustering algorithm to categorize the classification of cuproptosis based on the expression of the 10 CRGs, and three subtypes were identified **(** **Figure 2B** **)**. Cluster A included 259 cases, cluster B included 146 cases, and cluster C included 122 cases. Principal component analysis (PCA) further confirmed the differences between the three subtypes in transcription expression **(** **Figure 2C** **)**. The Kaplan–Meier curves revealed a shorter OS performance in patients with cluster C compared to the other two subtypes (Log-rank test, p=0.048; **Figure 2D**). Furthermore, we found that the main difference between the three clusters was the expression profiles of the GLS and CDKN2A genes **(** **Figure 2E** **).** Cluster C was characterized by significantly low expression of CDKN2A and high expression of GLS, suggesting that CDKN2A may serve as a tumor suppressor gene, while GLS may promote tumor progression. TCGA-LIHC cohort, which has complete clinical features and a large number of patients, was used to externally validate the repeatability of three cuproptosis subtypes. As we expected, three distinct clusters were classified and cluster C again showed the worthiest survival performance **(Log-rank test, p=0.0063;** **Figures 2F, G** **).**

### GSVA analysis and metabolic features of distinct cuproptosis subtypes

**Figure 3:**
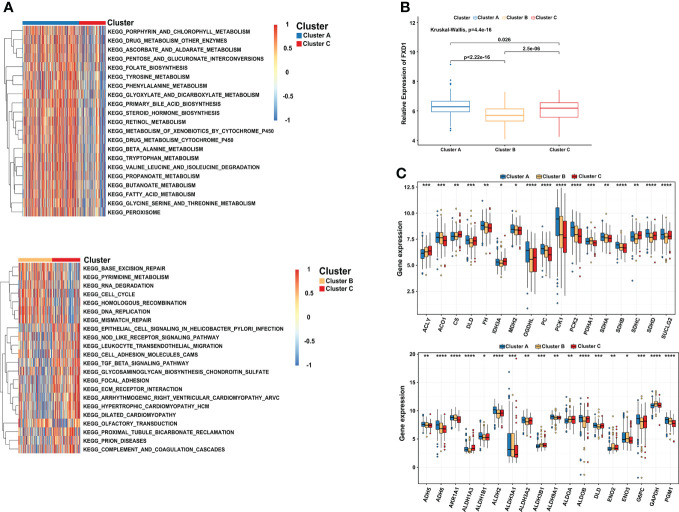
Comprehensive pathway enrichment analysis of three cuproptosis subtypes in the HCC patients. **(A)** GSVA analysis of three cuproptosis subtypes. Red and blue represent activated and inhibited pathways, respectively. **(B)** The association between FDX1 and distinct cuproptosis subtypes. **(C)** Boxplot revealing the expression of TCA_CYCLE pathways related genes (up) and GLYCOLYSIS_GLUCONEOGENESIS pathways related genes (down) in three cuproptosis subtypes. GSVA, gene set variation analysis. *p < 0.05; **p < 0.01; ***p < 0.001; ****p < 0.0001.

“Limma” and “GSVA” algorithms were performed to explore the potential biological functions in distinct subtypes. As the result showed, compared to subtype C, subtype A was significantly enriched in metabolism and biosynthesis pathways, including tyrosine, alanine, glyoxylate, and dicarboxylate metabolism. Among them, some metabolism pathways caught our attention, such as KEGG_METABOLISM_OF_XENOBIOTICS_BY_CYTOCHROME_P450 and KEGG_DRUG_METABOLISM_CYTOCHROME_P450 **(** **Figure 3A** **)**. As the previous study found that copper-dependent death occurs by interfering with the progress of the tricarboxylic acid (TCA) cycle (32), which means the metabolism of cytochrome p450 may be affected too. Subtype B was mostly involved in DNA replication, cell cycle, and mismatch repair pathway This may be explained by the higher expression of CDKN2A in subtype B, which is capable of inducing cell cycle arrest in the G1 and G2 phases. This also may be the reason why cluster B has the best survival advantage compared to cluster A. While subtype C has a rich TME, such as KEGG_CELL_ADHESION_MOLECULES_CAMS, KEGG_ECM_RECEPTOR_INTERACTION, KEGG_GLYCOSAMINOGLYCAN_BIOSYNTHESIS_CHONDROITIN_SULFATE, and KEGG_TGF_BETA_SIGNALING_PATHWAY. Comprehensive pathway enrichment analysis indicated that three cuproptosis subtypes have vital and distinct roles, respectively.

Since FDX1 participated in the metabolism and mitochondrial TCA cycle, additionally, cells based on mitochondrial respiration were more sensible of copper-induced cell death (33, 34). We further illuminated the association between FDX1 and distinct cuproptosis subtypes. Compared with the other two subtypes, cluster A indeed presented a significantly higher expression of FDX1 **(** **Figure 3B** **)**. More importantly, most of the genes involved in the TCA_CYCLE pathways were significantly upregulated in cluster A, such as OGDHL (Oxoglutarate Dehydrogenase L), PC (Pyruvate Carboxylase), PCK1 (Phosphoenolpyruvate Carboxykinase 1) and PCK2 (Phosphoenolpyruvate Carboxykinase 2). We observed a similar result in the GLYCOLYSIS_GLUCONEOGENESIS pathways, including ADH6 (Alcohol Dehydrogenase 6), ALDH2 (Aldehyde Dehydrogenase 2 Family Member), G6PC (Glucose-6-Phosphatase Catalytic Subunit), and PGM1 (Phosphoglucomutase 1) **(** **Figure 3C** **)**. The results showed that cluster A characterized by high expression of FDX1 was correlated with metabolism, glycolysis, and TCA cycle pathways, consistent with the previous research results (35, 36).

### Evaluation of immune characteristics and checkpoints in three cuproptosis subtypes

**Figure 4:**
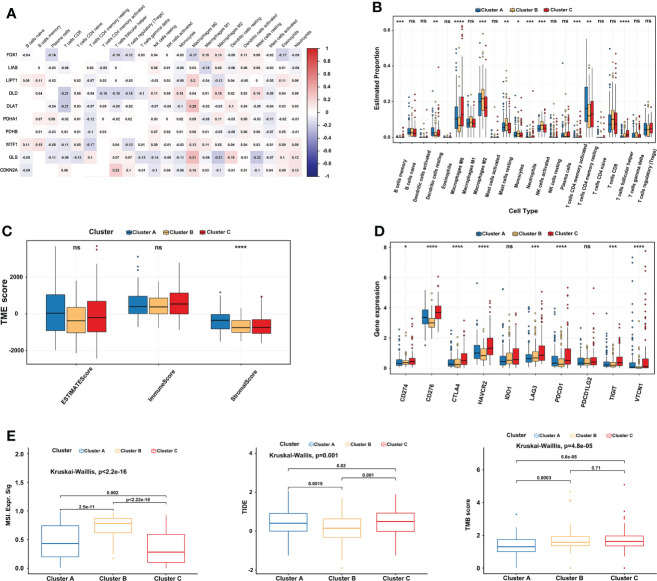
Comprehensive immune analysis of three cuproptosis subtypes in LIHC. **(A)** The correlation between 22 kinds of immune cells and 10 CRGs in TCGA-LIHC. Red represents positive interaction, blue represents negative interaction and the number in the brick represents Spearman’s correlation. **(B)** 22 infiltrating immune cell types abundance in three cuproptosis subtypes in TCGA-LIHC. **(C)** Stromal, immune, and estimate scores in three cuproptosis subtypes. **(D)** Expression levels of 10 immune checkpoints (including PD-1 and PD-L1) in three cuproptosis subtypes. **(E)** Relationships between three cuproptosis subtypes and MSI, TIDE score, and TMB. The Kruskal-Wallis test analyzed the statistical differences among the three subtypes. The asterisk represents the p-value (ns p > 0.05; *p < 0.05; **p < 0.01; ***p < 0.001). CRGs, cuproptosis-related genes; TCGA, The Cancer Genome Atlas; LIHC, liver hepatocellular carcinoma; MSI, microsatellite instability; TIDE, Tumor Immune Dysfunction and Exclusion; TMB, tumor mutational burden. ****p < 0.0001. ns, no significance.

10 CRGs were demonstrated to correlate with 22 kinds of immune cells using Spearman and CIBERSORT analysis in TCGA-LIHC **(** **Figure 4A** **)**. Subsequently, the immune cell infiltration of three cuproptosis subtypes was also explored. Compared to the other two clusters, the most significant immune infiltrating cells in cluster B were Macrophages M2, Mast cells resting, Monocytes, NK cells activated, and T cells CD8 **(** **Figure 4B** **)**. In addition, a high ImmuneScore was associated with cluster C, whereas cluster A had the highest score in the StromalScore. Although not significant, EstimateScore comprehensively showed that cluster B had the lowest score **(** **Figure 4C** **)**. Furthermore, we investigated the relationships between immune checkpoints and three cuproptosis subtypes. 7 immune checkpoints were differentially expressed in the different subtypes, including PD-1, CTLA-4, CD276, HAVCR2, LAG3, TIGIT, and, VTCN1. We found that cluster C usually had the highest expression in a total of 10 immune checkpoints, which indicates a potential better ICI therapy effect **(** **Figure 4D** **)**. After that, comprehensive analysis results showed significantly higher MSI and lower TIDE scores in cluster B, and there was no significant difference between clusters B and C in TMB scores **(** **Figure 4E** **)**, implying a possible benefit from ICI therapy.

### Establishment and validation of the CRGPI subgroups

**Figure 5:**
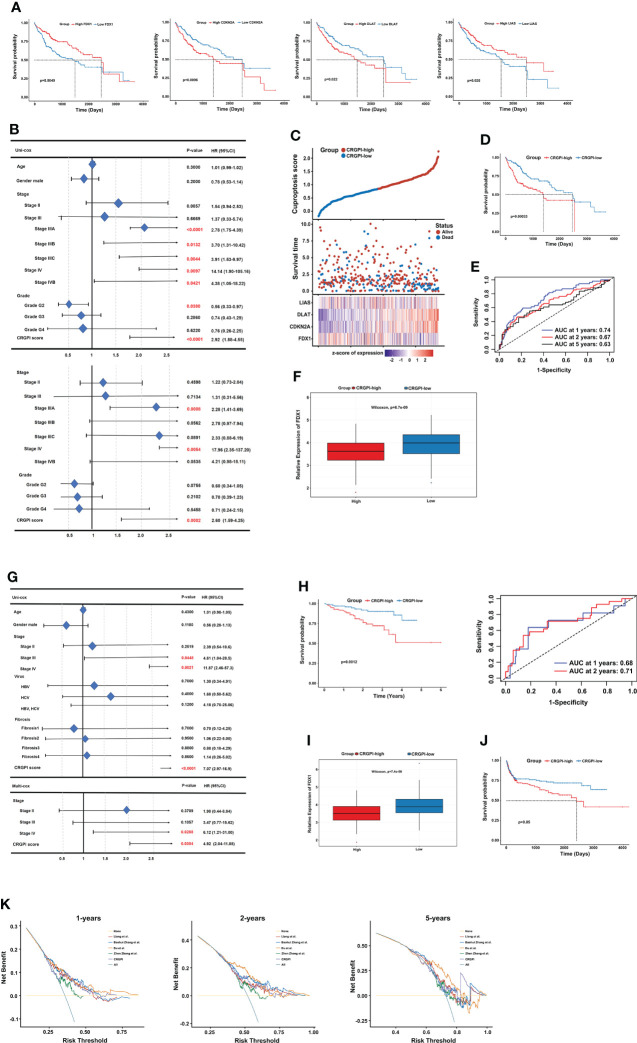
Prognostic analysis of different CRGPI score subgroups. **(A)** Kaplan–Meier curves of the four genes significantly in the univariate Cox analysis (log-rank test, all p < 0.05). **(B)** Univariate and multivariate Cox analysis of clinical factors and the CRGPI score factor in TCGA-LIHC. **(C)** Ranked scatter plots showing the distribution plot of the CRGPI score and patient survival events. **(D)** Kaplan–Meier analysis of the OS between the two subgroups (log-rank test, P = 0.00033). **(E)** ROC analysis at 1-, 2-, and 5-year survival according to the CRGPI score in the TCGA-LIHC cohort. **(F)** Boxplot showing the expression of FDX1 in different CRGPI subgroups in the TCGA-LIHC cohort. **(G)** Univariate and multivariate Cox analysis of clinical factors and the cuproptosis score factor in the ICGC-LIRI-JP cohort. **(H)** Kaplan–Meier and ROC curves of the OS between the two subgroups in the ICGC-LIRI-JP cohort (log-rank test, p = 0.0012). **(I)** Boxplot exhibiting the expression of FDX1 in different CRGPI subgroups in the ICGC-LIRI-JP cohort. **(J)** Kaplan–Meier survival analysis of the cuproptosis score subgroups in the HCC meta cohort (log-rank test, p = 0.05). **(K)** DCA curve assessing the clinical benefit of CRGPI with other four published molecular classifications at 1-, 2-, and 5-year PFS time. ROC, receiver operating characteristic; CRGs, cuproptosis-related genes; TCGA, The Cancer Genome Atlas; LIHC, liver hepatocellular carcinoma; OS, overall survival; DCA, decision curve analysis; CRGPI, cuproptosis-related gene prognostic index; PFS, progression free survival.

First, univariate Cox regression analysis was performed among the 10 CRGs to screen the independent prognostic genes, and four genes (FDX1, CDKN2A, DLAT, and LIAS) were significantly correlated with OS of patients in TCGA-LIHC **(** **Figure 5A** **)**. Then, a prognostic model was constructed based on the formula: CRGPI = expression level of FDX1*(-0.1393) + expression level of CDKN2A *(0.1746) + expression level of DLAT *(0.3614) + expression level of LIAS *(-0.125). Univariate Cox regression analysis showed that stage, grade, and CRGPI score were significantly associated with the prognosis of TCGA-LIHC, including clinicopathologic characteristics such as age and gender. Multivariate Cox regression analysis verified that stage and CRGPI score was indeed powerful prognostic factors **(** **Figure 5B** **)**. The distribution plot of the CRGPI indicated that survival times decreased with an increased CRGPI score **(** **Figure 5C** **)**. Taking the median CRGPI score as the cut-off value, the Kaplan–Meier survival curves revealed that CRGPI-high patients had a significantly worse OS than patients with a low score (p=0.00033, log-rank test; **Figure 5D**). Moreover, the 1-, 3-, and 5-year survival time of CRGPI scores were calculated by AUC values of 0.74, 0.67, and 0.63, respectively **(** **Figure 5E** **).** Since cuproptosis, also called copper-induced cell death, was attributable to Cu accumulation through FDX1-mediated protein lipoylation and destabilization of Fe–S cluster proteins, we explored the correlation between CRGPI subgroups and FDX1. As expected, the result showed that HCC patients in the CRGPI-high subgroup had a notably decreasing expression of pro-cuproptosis genes FDX1(p=6.7e-09; **Figure 5F**).

Furthermore, the role of the CRGPI was validated externally in ICGC-LIRI-JP with clinical factors, including age, gender, stage, virus, and fibrosis. The uni- and multi- COX analysis showed that the CRGPI was still a powerful predictive marker (HR= 7.074, p=0.0012; **Figure 5G**). The KM survival curve (p=0.0012, log-rank test) and ROC curve (0.68 at 1 year, and 0.71 at 2 years) also confirmed the patients in the CRGPI-high subgroup had a significantly worse OS compared with the low score subgroup **(** **Figure 5H** **)**. Consistent with the consequence of TCGA-LIHC, the ICGC-LIRI-JP patients in the CRGPI-high subgroup had a significantly lower expression of FDX1 **(** **Figure 5I** **)**. In addition, we specifically examined the predictive ability of the CRGPI in an HCC meta cohort, which had 527 patients with complete OS information, we could find a similar outcome with TCGA-LIHC and ICGC-LIRI-JP (p=0.05, log-rank test; **Figure 5J**). Taken together, these results demonstrated that HCC patients with high CRGPI scores had worse survival performance. Lower expression of FDX1 in the CRGPI-high subgroup may be the reason for worse OS because of less copper-induced tumor cell death. Decision curve analysis (DCA) proved that the CRGPI classification performed a great clinical net benefit compared with the other four molecular classifications strategies (Liang et al.; Baohui Zhang et al.; Du et al.; Zhen Zhang et al.) at 1-, 2-, and 5-year PFS time in the TCGA cohort **(** **Figure 5K** **)**.

### The benefit of ICI therapy in the CRGPI-high subgroup

**Figure 6:**
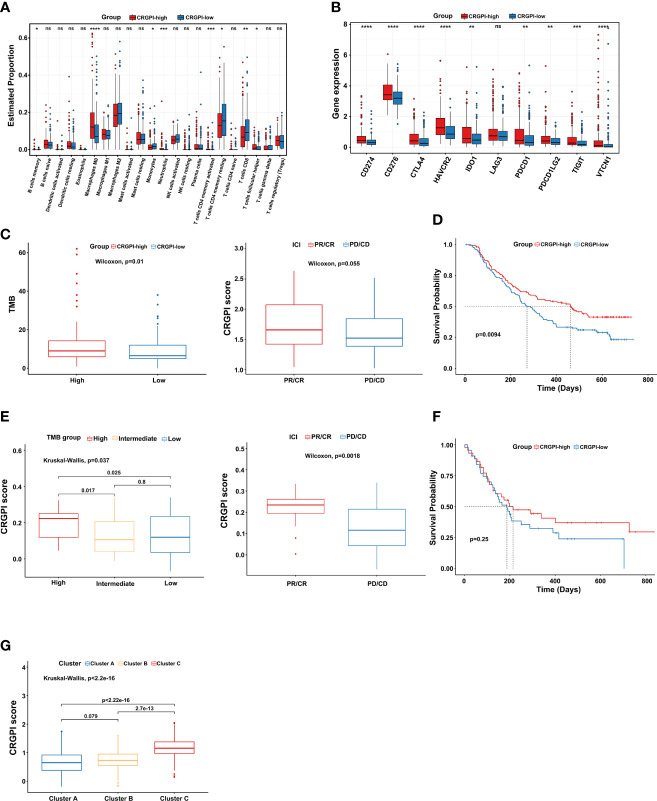
Comprehensive analysis of the CRGPI score. **(A)** Evaluation of the 22 immune cell types in CRGPI subgroups. **(B)** Expression of 10 immune checkpoints between the two subgroups. **(C)** The difference in TMB and immune response between two CRGPI subgroups in the IMvigor210 cohort. **(D)** Kaplan–Meier curve of different CRGPI subgroups for patients undergoing ICI therapy in the IMvigor210 cohort. **(E)** The difference in TMB and immune response between two CRGPI subgroups in the GSE176307. **(F)** Kaplan–Meier curve of different CRGPI subgroups for patients undergoing ICI therapy in the GSE176307. **(G)** The correlation between three cuproptosis subtypes and two CRGPI score subgroups. CRGPI, cuproptosis-related gene prognostic index; TMB, tumor mutational burden. ns, no significance.

First, we explored the immune cells infiltrating two CRGPI subgroups by performing the CIBERSORT algorithm. We observed that there were no significant differences in most immune cells, but the quantity of memory resting CD4+ T cells and CD8+ T cells was higher in the CRGPI-low group **(** **Figure 6A** **)**. Then, we investigated the associations between immune checkpoints and different CRGPI subgroups. In our results, the CRGPI-high group had higher immune checkpoints than the CRGPI-low subgroup (except for LAG3), implying that patients with high CRGPI scores were more likely to benefit from ICI therapy **(** **Figure 6B** **)**.

At present, anti-PD1/PD-L1 therapy plays an important role in ICI therapy. We further evaluated the prognostic ability of CRGPI in the IMvigor210 cohort and GSE176307 which both received ICI therapy. We found that patients in the CRGPI-high group had a significantly higher TMB and better response trend for ICI therapy in the Imvigor210 cohort. We as well observed that for those patients undergoing ICI therapy, the CRGPI-high subgroup had a better prognosis **(** **Figures 6C, D** **)**. As expected, results provided consistent evidence in the GSE176307 that patients with a higher CRGPI score were more likely to benefit from ICI therapy and associated with better OS **(** **Figures 6E, F** **)**. More interestingly, we observed a significant correlation between three cuproptosis subtypes and CRGPI score subgroups. The result showed that cluster C had the highest CRGPI score, indicating a high CRGPI score may be closely related to cluster C, which was characterized by better ICI therapy responses **(** **Figure 6G** **)**.

### Drug sensitivity analysis

**Figure 7:**
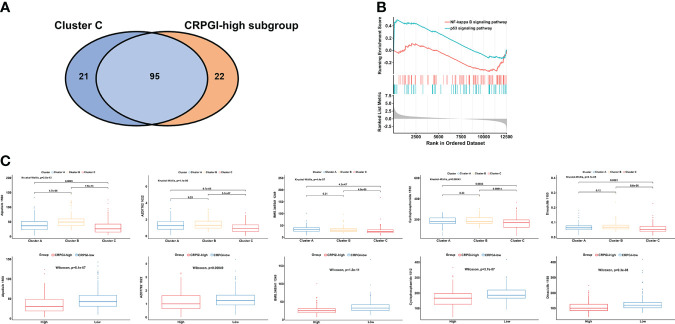
The correlation among three cuproptosis subtypes, two CRGPI subgroups, and drug sensitivity. **(A)** Venn plot displaying the intersect drugs by subtype C and CRGPI-high subgroup. **(B)** GSEA pathway enrichment of p53 and NF-kappa B signaling pathway in LIHC. **(C)** A comparison between the IC50 values of inhibitors related to the cell cycle and NF-κB signaling pathway in three cuproptosis subtypes (up), and two CRGPI subgroups (down). LIHC, liver hepatocellular carcinoma; gene set enrichment analysis (GSEA); CRGPI, cuproptosis-related gene prognostic index.

To study the correlation between cuproptosis and chemotherapy drugs in HCC, we assessed the IC50 values of 198 agents by performing a ridge regression algorithm in the GDSC2 database. First, we found that 116 drugs were significantly lower in cluster C compared to the other two cuproptosis subtypes. Similarly, the CRGPI-high subgroup had lower IC50 values of 117 drugs. Venn plot displayed that 95 agents were intersected, which implied that patients in the CRGPI-high subgroup or who belong to cluster C may benefit more from most kinds of chemotherapies **(** **Figure 7A** **).** In addition, we used GSEA to further explore the activity of pathway signaling in differential expressed genes of TCGA-LIHC and found that the activity of the p53 and NF-kappa B signaling pathways were upregulated in the HCC patients **(** **Figure 7B** **)**. Finally, we found that IC50 values of inhibitors related to the cell cycle signaling pathway, such as Alisertib_1051, AZD7762_1022, Dinaciclib_1180, Cyclophosphamide_1512 were significantly lower in the CRGPI-high group than in the CRGPI-low group. As we expected cluster C also showed significant results. Moreover, the results of BMS.345541_1249 indicated that patients with high risk or cluster C may benefit more from the IκB/IKK inhibitor **(all P < 0.05,** **Figure 7C** **)**. Together, cuproptosis were related to chemotherapy drug sensitivity in HCC.

## Discussion

The results of this study revealed genetic alterations and transcriptional expression levels of 10 CRGs in LIHC. We identified three cuproptosis subtypes based on 10 CRGs and found patients with subtype C had worse OS. High expression of GLS, while low expression of CDKN2A of subtype C may explain the poor prognosis. GLS (Glutaminase), which is also known as the “kidney-type” glutaminase (GLS1), is a metabolism enzyme that plays a critical role in glutaminolysis that promotes cancer cell proliferation, including HCC. Moreover, HCC is addicted to glutamine, which means GLS is often overexpressed in hepatocellular cancer cells to fulfill enhanced energy demand (37, 38). The inhibitors of GLS in cancer Therapy worked by interfering with the metabolism of alpha-ketoglutarate, an intermediate of the tricarboxylic acid (TCA) cycle, some of which are undergoing clinical trials and exhibiting promising effects (39, 40). Previous studies found that the tumor suppressor gene CDKN2A (Cyclin Dependent Kinase Inhibitor 2A) encodes p16INK4a and p14ARF (41). P16INK4a inhibits cell-cycle progression from G1 to S phase by CDK4/6-mediated phosphorylation of retinoblastoma protein (Rb) (42). P14ARF stabilizes the function of the tumor suppressor gene p53 by inhibiting its degradation. Loss of CDKN2A causes inactivation of the Rb and p53 pathways, generating uncontrolled cell proliferation (43). Moreover, according to previous studies, the loss or mutating of CDKN2A causes uncontrolled cancer cell proliferation, and TP53 mutations are correlated with CDKN2A mutation and high TMB (44, 45). In our study, we found that CDKN2A had a high mutation rate in HCC, which was consistent with the previous study showed that CDKN2A is frequently mutated or deleted in a wide variety of tumors.

Understanding the metabolic features and immune cell infiltration characteristics in the TME among distinct cuproptosis subtypes could help in identifying the different molecular and immune patterns in HCC. We found that subtype B was significantly characterized by cell cycle-related pathways and that may be due to the high expression of CDKN2A. Subtype C had the most complicated microenvironment, featured with riched ECM, glycosaminoglycan, and focal adhesion. Notably, subtype A is mostly characterized by kinds of metabolism and biosynthesis pathways, including the metabolism of cytochrome p450 and amino acids. Additionally, we also found that the expression of FDX1, TCA_CYCLE pathways-related genes, and GLYCOLYSIS_GLUCONEOGENESIS pathways-related genes were associated with subtype A. Those results indicated that cluster A was correlated with metabolism, glycolysis, and TCA cycle pathways. High expression of FDX1 in subtype A may be one of the reasons (35, 36).

Then, we found that immune cell infiltration was significantly related to cuproptosis subtypes, and explored the relationship between three cuproptosis clusters and known predictive biomarkers for immunotherapy, including multiple immune checkpoints (PD-1, PD-L1, etc…), TIDE, MSI, and TMB. Our results showed patients in subtype B with MSI-high also had low TIDE, but cluster C had the highest TMB, and there is no significant difference in TMB between clusters B and C. In general, a consistent result suggests that subtypes B and C may be the immunotherapy-response phenotypes.

Considering the heterogeneity of HCC, we calculated an accurate CRGPI score for every patient to guide personalized therapy and divided patients into two subgroups according to the median score as a cutoff value (46, 47). The CRGPI signature was constructed based on 4 genes (FDX1, CDKN2A, DLAT, and LIAS). FDX1(Ferredoxin 1) functions as the key gene for the progress of cuproptosis by reducing cupric ions to cuprous ions releasing them into the mitochondrial matrix and works as an upstream regulator in the process of protein lipoylation, thus disturbing the progress of TCA (tricarboxylic acid) cycle and in the TCA cycle (35, 48). DLAT (Dihydrolipoamide S-Acetyltransferase) encodes part of the pyruvate dehydrogenase (PDH) complex (PDC) component, which is associated with pyruvate metabolism in the TCA cycle (49). LIAS (Lipoic Acid Synthetase) is an enzyme-containing two [4Fe-4S] clusters and has been linked to lipoic acid metabolism.

Recently, studies have reported that some biomarkers could predict the prognosis of HCC. Liang et al. reported a 10 ferroptosis-related gene signature for predicting OS in HCC. Baohui Zhang et al. established a hypoxia-related signature based on three genes (PDSS1, CDCA8, and SLC7A11) for predicting diagnosis, prognosis, and immune microenvironment of HCC. Du et al. built a seven-mRNA biomarker based on microvascular invasion (MVI) to predict the recurrence of HCC. Recently, Zhen Zhang et al. identified cuproptosis-related risk score to predict prognosis and characterized the TME of HCC. Decision curve analysis (DCA) is a useful tool to assess the utility of different models for clinical decision-making (26). The analysis proved that the CRGPI classification performed a great clinical net benefit compared with the other four molecular classifications strategies at 1-, 2-, and 5-year PFS time in the TCGA-LIHC cohort. Here, our results implied that CRGPI was a powerful prognostic biomarker and demonstrated that HCC patients with high CRGPI scores had worse survival performance. Lower expression of FDX1 in the CRGPI-high subgroup may be the reason because of less copper-induced tumor cell death, consistent with our previous FDX1 survival results.

Next, we found that cluster C had the highest CRGPI score and the CRGPI-high subgroup had a similar TME as cluster C. For most immune cell infiltration, there were no significant differences except for the quantity of memory resting CD4+ T cells and CD8+ T cells. In addition, we found that the CRGPI-high group had higher immune checkpoints expression than the CRGPI-low subgroup, implying that patients with high CRGPI scores were more likely to benefit from ICI therapy. We further validated that the CRGPI-high group had high expression of PD1/PDL1, TMB, and better response (PR/CR) to immunotherapy in two anti-PD1/PD-L1 therapy cohorts (IMvigor210 cohort and GSE176307). Taken together, those data indicated that, for HCC patients who have undergone ICI therapy, the CRGPI-high subgroup was more likely to benefit from ICI therapy and had a better prognosis.

Interestingly, our results suggested that HCC patients with high CRGPI scores may have a worse OS probability. However, they may also be sensitive to ICI therapy and would have a better survival outcome if choosing anti-PD1/PD-L1 treatment, compared with the CRGPI-low subgroup. These findings highlight the importance of CRGPI serving as a potential biomarker for both prognostic and immunotherapy for HCC patients. The decreasing expression of the pro-cuproptosis gene FDX1 in the CRGPI-high subgroup may explain the plausible mechanism for the correlation between high CRGPI scores and their responsiveness to ICI therapies. FDX1, a key gene involved in cuproptosis, could influence metabolism function and regulate the mitochondrial enzymes of the TCA (48, 50). According to previous studies, Naive T cells generate energy by oxidative phosphorylation (OXPHOS) and switch their metabolic pattern to glycolysis once activated. Besides, sufficient supply of glucose and glutamine are vital metabolite nutrition required for T cell differentiation and function. However, cancer cells would compete for glucose and glutamine intake from T cells to enhance their growth and proliferation (51–53). ICI therapy is based on the properties of T cells targeting therapeutic checkpoints, such as PD1. We hypothesized that the CRGPI-high subgroup with a low expression of FDX1 may indicate less metabolism intake from T cells and such effects might help enhance the effect of immunotherapy.

In summary, this study systematically analyzed CRGs in LIHC, and our comprehensive analysis demonstrated that (i). The landscape of molecular characteristics of the three cuproptosis subtypes. (ii). The CRGPI score may serve as a promising prognostic biomarker and help in distinguishing potential immunotherapy effective patients. But further clinical research is needed to confirm our study. Generally, our results provide novel insights about cuproptosis into immune therapeutic strategies.

## Conclusion

In conclusion, our comprehensive analysis may help us understand the molecular characteristics based on CRGs in HCC. Besides, the CRGPI score may serve as a potential prognostic biomarker and guide personalized molecular targeted therapy and ICI therapy.

## Data availability statement

The original contributions presented in the study are included in the article/**Supplementary Material**. Further inquiries can be directed to the corresponding authors.

## Author contributions

LD participated in designing and preparing the manuscript. WL, JT, ZC, and JiL participated in data analysis. HC, JuL, and YL participated in all figures and table preparation. QY and GL participated in the steam-processed. All authors contributed to the article and approved the submitted version.

## Conflict of interest

The authors declare that the research was conducted in the absence of any commercial or financial relationships that could be construed as a potential conflict of interest.

## Publisher’s note

All claims expressed in this article are solely those of the authors and do not necessarily represent those of their affiliated organizations, or those of the publisher, the editors and the reviewers. Any product that may be evaluated in this article, or claim that may be made by its manufacturer, is not guaranteed or endorsed by the publisher.

## Supplementary material

Supplementary Figure 1 Enrolled datasets in this study. **(A)** The flowchart of HCC mRNA datasets exclusion criteria. **(B)** The flowchart of two anti-PD1/PD-L1 therapy cohorts (IMvigor210 cohort and GSE176307) exclusion criteria.
