# Supplementary figures and images for: Identification of cuproptosis-related subtypes, cuproptosis-related gene prognostic index in hepatocellular carcinoma
Source: Front Immunol. 2022 Sep 13;13:989156. doi: 10.3389/fimmu.2022.989156 (PMC9513033; doi:10.3389/fimmu.2022.989156)

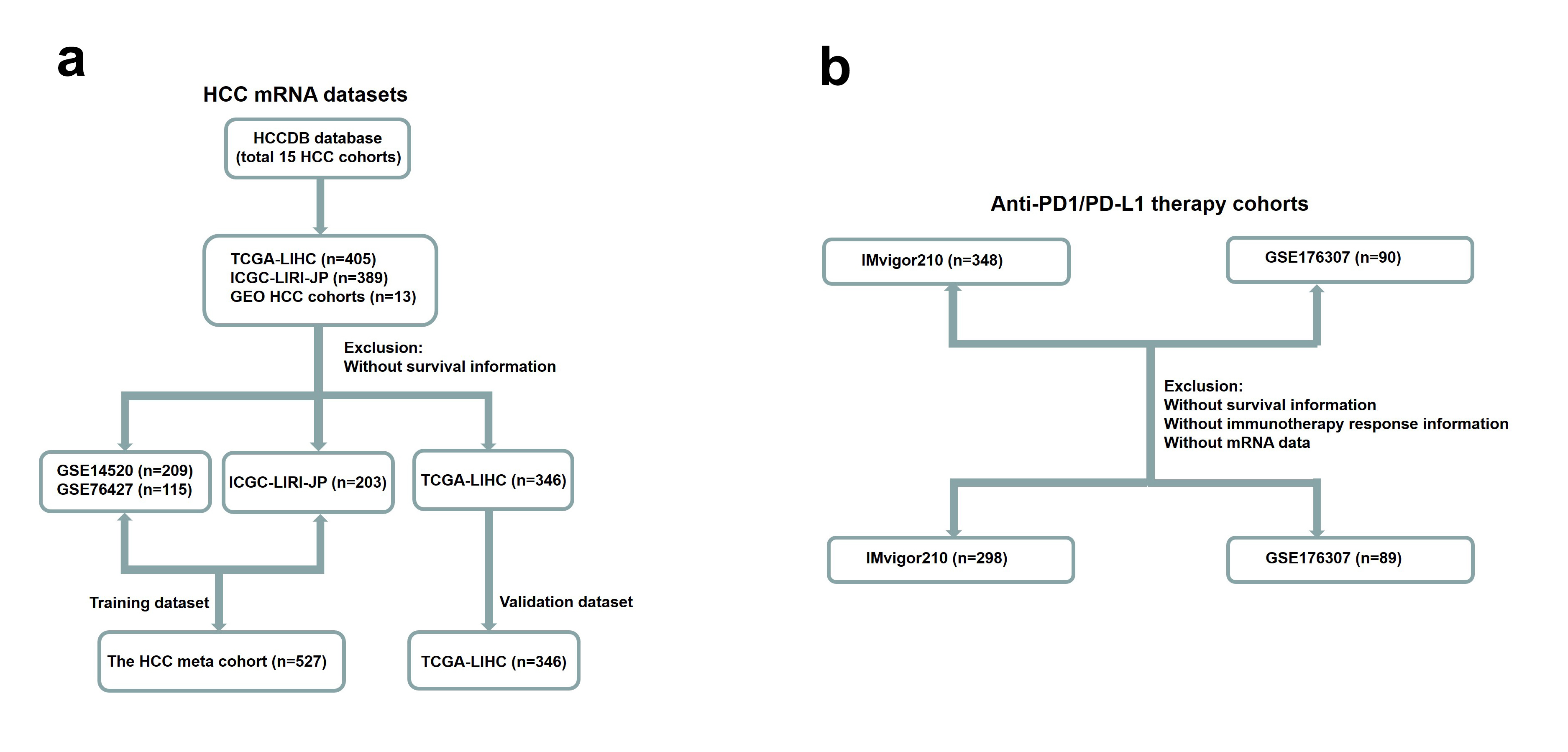

Supplement: Supplementary Figure 1 — Enrolled datasets in this study. (A) The flowchart of HCC mRNA datasets exclusion criteria. (B) The flowchart of two anti-PD1/PD-L1 therapy cohorts (IMvigor210 cohort and GSE176307) exclusion criteria. [file Image_1.jpeg]
